# Rationale and design of the balANZ trial: A randomised controlled trial of low GDP, neutral pH versus standard peritoneal dialysis solution for the preservation of residual renal function

**DOI:** 10.1186/1471-2369-11-25

**Published:** 2010-09-16

**Authors:** David W Johnson, Margaret Clarke, Vanessa Wilson, Feidhlim Woods, Fiona G Brown

**Affiliations:** 1 Department of Renal Medicine, Princess Alexandra Hospital, Brisbane, Australia; Southern; 2 School of Medicine, University of Queensland, Brisbane, Australia; 3 Fresenius Medical Care, Australia Pty Ltd; 4 Fresenius Medical Care Asia-Pacific, Hong Kong; 5 Department of Nephrology, Monash Medical Center, Clayton, Victoria, Australia; 6 for the balANZ Trial Study Group

## Abstract

**Background:**

The main hypothesis of this study is that neutral pH, low glucose degradation product (GDP) peritoneal dialysis (PD) fluid better preserves residual renal function in PD patients over time compared with conventional dialysate.

**Methods/Design:**

Inclusion criteria are adult PD patients (CAPD or APD) aged 18-81 years whose first dialysis was within 90 days prior to or following enrolment and who have a residual GFR ≥ 5 ml/min/1.73 m^2^, a urine output ≥ 400 ml/day and an ability to understand the nature and requirements of this trial. Pregnant or lactating patients or individuals with an active infection at the time of enrolment, a contra-indication to PD or participation in any other clinical trial where an intervention is designed to moderate rate of change of residual renal function are excluded. Patients will be randomized 1:1 to receive either neutral pH, low GDP dialysis solution (Balance^®^) or conventional dialysis solution (Stay.safe^®^) for a period of 2 years. During this 2 year study period, urinary urea and clearance measurements will be performed at 0, 3, 6, 9, 12, 18 and 24 months. The primary outcome measure will be the slope of residual renal function decline, adjusted for centre and presence of diabetic nephropathy. Secondary outcome measures will include time from initiation of peritoneal dialysis to anuria, peritoneal small solute clearance, peritoneal transport status, peritoneal ultrafiltration, technique survival, patient survival, peritonitis rates and adverse events. A total of 185 patients has been recruited into the trial.

**Discussion:**

This investigator-initiated study has been designed to provide evidence to help nephrologists determine the optimal dialysis solution for preserving residual renal function in PD patients.

**Trial Registration:**

Australian New Zealand Clinical Trials Registry Number: ACTRN12606000044527

## Background

During the past 30 years, peritoneal dialysis (PD) has become an established form of treatment for patients with end-stage renal disease [1,2]. Most published observational cohort studies suggest that the medium-term survival (up to 3 to 4 years) of patients treated with PD is at least comparable, and possibly superior, to that of patients receiving haemodialysis (HD) [3-7]. However, PD is associated with a higher rate of technique failure than HD. As rates of peritonitis have fallen in recent years, an increasing proportion of technique failure is attributable to alteration in membrane function whereby high solute transport is associated with loss of ultrafiltration capacity [8]. Unfortunately, the time sequence of loss of peritoneal ultrafiltration capacity and loss of residual renal function and urine output coincide whereby the two significantly limit an individual patient's capacity to control solute and volume homeostasis.

A large body of basic research in animal models and peritoneal cell culture systems has suggested that a major contributor to the high technique failure rate is the bioincompatible nature of conventional PD fluids. Such fluids may have a negative impact on host defence as well as having a pro-fibrotic effect on the peritoneal membrane [9-11]. Conventional PD fluids are considered "unphysiological," based on their acidic pH (5.0-5.8), high lactate concentrations (30-40 mmol/L), high osmolality (320-520 mOsm/kg), high glucose concentrations (31-236 mmol/L), and contamination by glucose degradation products (GDP) generated during the heat sterilization process [12]. Such solutions reduce the viability and growth of peritoneal mesothelial cells and fibroblasts in vitro, alter the turnover of structural collagen, and modify the homeostatic balance of cytokines and growth factors [11,13,14]. The viability and function of peritoneal phagocytic cells, such as macrophages, are also impaired by standard peritoneal fluids [12,13]. Moreover, experimental and clinical exposure of the peritoneal membrane to conventional PD solutions engenders significant histopathological changes over time, including loss of the surface mesothelial cell layer, thickening of the submesothelial compact zone and the development of a progressive vasculopathy [15,16]. Most of these adverse effects of dialysate on the peritoneal membrane appear to be accounted for by acidic pH and high concentrations of GDPs, since they were largely abrogated in *in vivo *studies by the use of neutral-buffered, low GDP fluids [11,12,17,18]. In addition to their direct cytotoxicity and stimulation of inflammatory cytokine production, GDPs promote the formation and deposition of advanced glycation end-products (AGEs) in the peritoneal membrane, which in turn correlates with peritoneal membrane fibrosis and histopathology [19,20].

All of the main industry manufacturers of PD solutions have now released to the market solutions that have low GDP levels (and, incidentally, a neutral or near neutral solution pH). Recent clinical trials with these solutions attest to their lower cytotoxicity for mesothelial cells and their use is also associated with less evidence of local peritoneal inflammation [21,22]. However, evidence of a beneficial effect on the morphological and functional changes associated with long-term exposure to PD fluids is not yet available. Nevertheless, a large, retrospective, observational cohort study in Korea [23,24] has demonstrated an association between the use of neutral pH, lactate-buffered, low GDP fluids and superior survival, although this finding was potentially limited by indication bias with residual confounding [25].

An alternative means by which the use of neutral pH, low GDP solutions might favourably impact PD patient outcomes relates to improved preservation of native kidney function. In the literature relating to survival of PD patients, there is overwhelming evidence that survival is determined more by residual renal function than by peritoneal function [26-28]. A multi-centre, open-label, prospective, randomized crossover study of conventional, acidic, lactate-buffered fluid with pH neutral, lactate-buffered, low GDP fluid in 86 prevalent CAPD patients from 22 centres in 11 European countries [22] demonstrated increases in renal urea and creatinine clearances over a 12 week period. Subsequently, several small, short-term, randomised controlled trials reported that neutral pH, low GDP fluids were associated with either a neutral [29,30] or beneficial [31] effect on residual renal function. However, these studies were limited by insufficient statistical power, short-term follow-up and single centre designs.

The current multi-centre, randomised controlled study is designed to ask whether neutral pH, low GDP peritoneal dialysis fluid better preserves residual renal function in PD patients over a 2 year period compared with conventional dialysate.

## Methods/Design

Ethics approval for the balANZ trial has been obtained from the local Institutional Ethics Committee in all participating centres prior to study initiation and patient enrolment. The study will be performed in accordance with the 2000 Edinburgh, Scotland Revision of the Declaration of Helsinki, the National Health and Medical Research Committee (NHMRC) Statement on Human Experimentation, Joint NHMRC/AVCC Statement and Guidelines on Research Practice, applicable ICH guidelines and the Therapeutic Goods Administration (TGA) - Note for guidance on good clinical practice (CPMP/ICH/135/95) annotated with TGA. All patients are to provide written informed consent before any trial related procedure can occur.

### Participants

Patients must fulfill all of the following conditions in order to be considered for the study enrolment or participation:

• Male or female patients, age ≥ 18 years and < 81 years

• Diagnosis of end stage renal disease

• First treatment for ESRD by any dialysis modality within 90 days prior to or following enrolment (patients may be enrolled prior to commencing first treatment if there is clear indication that the treatment modality is CAPD or APD and they consent in advance to enter the study)

• Selected to be treated by CAPD or APD

• Residual GFR at enrolment ≥ 5 ml/min/1.73 m^2^

• Urine volume per day ≥ 400 ml at enrolment

• Written informed consent before any trial related activities

• Ability to understand the nature and requirements of this trial

Patients with any of the following conditions will be excluded from study enrolment:

• Prognosis for survival less than 12 months

• Pregnancy or lactation period

• History of malignancy other than a successfully and completely treated cutaneous squamous cell or basal cell carcinoma or carcinoma in-situ of the cervix within the last 5 years

• Any acute infections at the time of enrolment into the study

• Any disease of the abdominal wall, such as injury or surgery, burns, hernia, dermatitis, that in the opinion of the Investigator would preclude the patient from being able to have peritoneal dialysis

• Any inflammatory bowel diseases (Crohns' disease, ulcerative colitis or diverticulitis) that in the opinion of the Investigator would preclude the patient from being able to have peritoneal dialysis

• Any intra-abdominal tumours or intestinal obstruction

• Any patient with active serositis

• Any condition (mental or physical) that would interfere with the patient's ability to comply with the study protocol

• Known or suspected allergy to trial product or related products

• Participation in any other clinical trial where an intervention is designed to moderate rate of change of residual renal function

### Study Design

**Figure 1:**
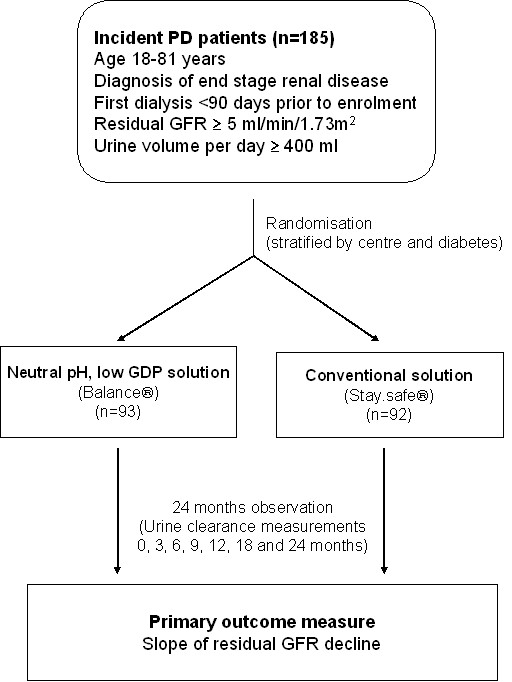
**Schema for the balANZ Trial**.

The study is an investigator-initiated, prospective, open label, randomized, placebo-controlled phase 4 trial. Patients will be randomised to one of two treatment groups in equal proportion (Fig. 1). To ensure adequate concealment of allocation, the randomization will be performed using a central computer and web-based link to the central database. Stratification will occur according to centre and the presence or absence of diabetes mellitus. Patients will be recruited from 16 centres across Australia, New Zealand and Singapore.

### Experimental Intervention

Patients in the experimental arm will receive neutral pH, lactate-buffered, low GDP *Balance*^® ^solutions in Biofine^®^, platicizer-free solution bags provided by Fresenius Medical Care. The chemical composition of this fluid is shown in Table 1.

**Table 1:** Comparison of the chemical composition of the experimental (Balance^®^) and control (Stay.safe^®^) fluids used in the balANZ trial

Component	Experimental (Balance^®^)	Control (Stay.safe^®^)
Sodium (mmol/L)	134	134

Chloride (mmol/L)	100.5	102.5

Lactate (mmol/L)	35	35

Bicarbonate (mmol/L)	2	0

pH	7	5.5

Glucose (g/L)	15-42.5	15-42.5

3-deoxyglucosone (μmol/L)	42-60	173-324

Methylglyoxyl (μmol/L)	< 1	6-10

Acetaldehyde (μmol/L)	< 2	152-182

Formaldehyde (μmol/L)	< 3	7-13

Calcium (mmol/L)	1.25	1.25

Magnesium (mmol/L)	0.5	0.5

### Control Intervention

Patients in the control arm will receive conventional, standard, lactate-buffered PD solutions (Stay.safe^®^) in Biofine^®^, platicizer-free solution bags provide by Fresenius Medical Care. The chemical composition of this fluid is shown in Table 1.

### Concurrent Treatments

Patients in each trial arm will undergo standard management, as per local PD unit protocols. Icodextrin is permitted in both the control and experimental groups.

### Blinding

This is an open-label study. Therefore, after randomization, the investigator, pharmacist and patient will be aware of which peritoneal dialysis solution the patient will be receiving.

### Outcome Measures

The primary outcome measure is slope of residual renal function (RRF) decline measured as GFR (mean of renal urea and creatinine clearances) over time (follow-up 24 months).

Secondary outcomes will include:

a. Time from initiation of PD to anuria (daily urine volume less than 100 ml).

b. Peritoneal small solute clearance (Kt/V, creatinine clearance)

c. Peritoneal transport status (PET D/P creatinine and D/Do glucose)

d. Peritoneal ultrafiltration capacity (UF[ml/day] and nUF[ml/day/m^2^])

e. Technique survival

f. Patient survival

g. Peritonitis rates

h. Adverse events

### Clinical Assessment of Outcome

Residual renal function will be measured from timed urine collections as the arithmetic mean of urinary urea and creatinine clearance [32]. Data will be collected at the start of the study-phase (baseline week 0, visit 0), and at Month 3 and then at 3 monthly intervals thereafter (total 6 measurements) in the first 12 months. Data will be collected at 6 monthly intervals if treatment extends into the second year. A maximum of 8 visits will be required for the study. Visit windows will be ± 1 week in the first year and ± 2 weeks in the second year of treatment.

### Monitoring for Adverse Events

The number and proportion of subjects who report treatment-emergent adverse events will be summarized for each treatment group. Treatment emergent events include events that start on or after Day 0 of the study [that is the first day of Study Drug administration], and were not present at baseline, or were present at baseline, but increased in severity after the start of the study. The Medical Dictionary for Regulatory Activities [MedDRA] Terminology will be used to classify all adverse events with rESAect to System Organ Class [SOC], high level group term (HLGT), and preferred term. *Balance*^® ^and stay*safe^® ^solutions are already registered and marketed PD solutions in terms of their composition and intended use.

### Sample Size Calculations

A prospective sample size calculation was based on information collected from earlier phase work, with an anticipated rate of decline of residual renal function (RRF) with Balance at -0.044 ml/min/1.73 m^2^/month compared with stay safe at -0.111 ml/min/1.73 m^2^/month, with a common standard deviation of 0.219 ml/min/1.73 m^2^/month. The estimated sample size per arm based on this information was 168 (total population 336), giving the study 80% power to detect a difference in slope of RRF versus time of 0.067.

### Trial Completion

Commencing in November 2004, it was initially anticipated that trial recruitment would be completed by December 2008, with final follow-up completed by December 2010. As of 1 October 2008, only 185 patients had been recruited into the study, accounting for 55% of the target. A decision was therefore made by the Trial Management Committee to halt further recruitment and to continue the trial until the last patient enrolled had been followed for 2 years (31 August 2010). Results will be analysed and reported in early 2011. Based on the final number recruited (185), the study has 80% power to detect a difference in RRF versus time of 0.091 ml/min/1.73 m^2^/month.

### Statistical Analyses

A mixed effects General Linear Model will be fitted with residual renal function as the outcome variable and treatment group, time, centre and presence of diabetic nephropathy (Yes/No) as fixed effects terms. Patients will be fitted as a 'random' term in the model. In this way, the model will provide estimates of the rate of decline in RRF (the slope) for each patient and an appropriate framework to model the covariance structure for repeated measures. From this, an overall estimate of the rate of decline in each treatment group will be determined, corrected for centre and presence of diabetic nephropathy. The estimated slopes for the treatment groups will be compared via a t-test. The data will be assumed to be normally distributed and to decline in a linear fashion. However, the assumptions of homogeneity of variance and normality of residuals will be checked. Major departures from these assumptions will result in the employment of appropriate non-parametric tests or transformation. This analysis will be performed for both the month 12 and month 24 reporting efforts on an intention-to-treat basis. Secondary time to event analyses will be analysed by multivariate Cox proportional hazards model analyses with treatment group, centre, presence of diabetic nephropathy and baseline renal function as covariates. P values < 0.05 will be considered significant.

## Discussion

This investigator-initiated, multicentre Australian, New Zealand and Singapore study has been designed to provide evidence to help nephrologists and their PD patients better determine the optimal dialysis solution for preserving residual renal function. Given that numerous studies have demonstrated that PD patients with rapid residual renal function decline are at significantly increased risk of mortality [26-28], novel treatments for preserving residual renal function in this group (such as with the use of neutral pH, low GDP dialysis fluids) may represent an important strategy for improving clinical outcomes in PD patients. The multicentre nature of the trial will greatly enhance its generalisability.

One of the significant difficulties encountered to date with the running of the trial has been slower than anticipated recruitment. Some of the reasons for this included initial delays with being able to accommodate patients receiving APD as well as a reluctance by some clinicians to allow patients to be randomised to conventional dialysate because of a firmly held belief that such fluids were inferior to "biocompatible" fluids. The hurdle encountered in recruiting to target in the balANZ trial due to the fact that use of biocompatible fluids is becoming standard in Australasia and many other parts of the world without high level supporting evidence, suggest that it may be extremely difficult for a future larger randomised controlled trial to be mounted if the present study does not prove to be definitive. Nevertheless, it is extremely important to establish high level clinical evidence for the benefits of "biocompatible" fluids compared to conventional dialysates, since they are appreciably more expensive and consequently have important implications for the allocation of limited health resources. Simply relying on biological plausibility and the results of observational cohort studies is fraught with peril due to confounding by indication and other biases. Indeed, there are numerous examples in nephrology practice where firm clinical guideline recommendations based on opinion supported by observational studies have been subsequently over-turned by the findings of randomised controlled clinical trials and systematic reviews (viz. peritoneal dialysis small solute clearance augmentation, serum cholesterol lowering and anaemia correction).

Although the final balANZ trial population will be just over half that required to provide adequate statistical power according to prospective sample size calculations, it will nevertheless be the largest randomised controlled clinical trial to date of "biocompatible" versus conventional dialysis fluids in PD patients and will be twice the size of the next largest, published controlled trial [29]. Whilst the reduced final sample size of 185 will ultimately result in decreased statistical power, the study will still have 80% power to detect a difference in RRF decline of 0.091 mL/min/1.73 m^2^/month. This detectable difference compares favourably with the differences in RRF decline reported between Balance^® ^and Stay.safe^® ^fluids in other trials, such as Euro-Balance (0.21 mL/min/1.73 m^2^/month over 3 months) [22] and Balnet (0.80 mL/min/1.73 m^2^/month over 12 months) [31]. In the event of a negative study finding, which might potentially reflect a type 2 statistical error, the ability to detect a smaller benefit of biocompatible fluids on residual renal function decline could still be possible as a result of incorporation of the balANZ trial data into a meta-analysis of all randomised controlled trials of biocompatible fluids versus conventional biocompatible fluids currently being undertaken by the Cochrane Renal Group [33]. Such a systematic review may also help to determine whether biocompatible fluids influence patient-level outcomes, such as technique survival and patient survival, for which all studies to date have been significantly underpowered.

In conclusion, demonstration by the balANZ study of a significant improvement in residual renal function decline with neutral pH, low GDP fluids will provide clinicians with an important new strategy for effectively treating PD patients. On the other hand, a negative study will dissuade clinicians from prescribing more expensive fluids for no clear clinical benefit.

## Abbreviations

balANZ: the Balance in Australian and New Zealand peritoneal dialysis patients trial; D/D_0 _glucose: ratio of initial and 4 hour dialysate glucose concentrations; D/P creatinine: dialysate to plasma creatinine ratio at 4 hours; ESRD: end-stage renal disease; GFR: glomerular filtration rate; HD: Haemodialysis; ICH: International Conference on Harmonisation; NHMRC: National Health and Medical Research Council; nUF: normalised ultrafiltration; PAH: Princess Alexandra Hospital; PET: peritoneal equilibration test; RRF: residual renal function; TGA: Therapeutic Goods Administration; UF: ultrafiltration.

## Competing interests

David Johnson is a consultant for Baxter Healthcare Pty Ltd and has previously received research funds from this company. He has also received speakers' honoraria and research grants from Fresenius Medical Care. Fiona Brown is a consultant for Baxter and Fresenius. Margaret Clarke and Vanessa Wilson are employees of Fresenius Medical Care. Feidhlim Woods is a former employee of Fresenius Medical Care and is currently a consultant to this company.

The balANZ trial is funded by Fresenius Medical Care.

## Authors' contributions

DJ and FB were the principal investigators; conceived study; participated in design and co-ordination; helped to draft manuscript; read and approved the final manuscript. MC participated in co-ordination; helped to draft manuscript; read and approved the final manuscript. SG participated in co-ordination; helped to draft manuscript; read and approved the final manuscript. VW participated in design and co-ordination; helped to draft manuscript; read and approved the final manuscript. FW participated in design and co-ordination; helped to draft manuscript; read and approved the final manuscript.

## Pre-publication history

The pre-publication history for this paper can be accessed here:

http://www.biomedcentral.com/1471-2369/11/25/prepub
